# Birth weight, family history of diabetes and diabetes onset in schizophrenia

**DOI:** 10.1136/bmjdrc-2019-001036

**Published:** 2020-01-28

**Authors:** Emilio Fernandez-Egea, Ryan Walker, Hisham Ziauddeen, Rudolf N Cardinal, Edward T Bullmore

**Affiliations:** 1 Department of Psychiatry, Behavioural and Clinical Neuroscience Institute, University of Cambridge, Cambridge, UK; 2 Cambridgeshire and Peterborough NHS Foundation Trust, Cambridge, UK; 3 Wellcome Trust-MRC Institute of Metabolic Science, Cambridge, UK

**Keywords:** clozapine, development, psychiatry

## Abstract

**Introduction:**

The prevalence of diabetes in schizophrenia is twice that in the general population, but there are few reliable predictors of which individuals will develop glucose dysregulation.

**Objective:**

To test if abnormal birth weight (either too low or too high) and parental diabetes, both variables that can be ascertained in the clinic, can predict diabetes onset in patients with schizophrenia.

**Research design and methods:**

Electronic records of a cohort of 190 clozapine-treated patients (37% treated for more than 20 years) and Cox regression survival analysis (with any type of glucose dysregulation as the event) to account for differences in length of treatment before the event and age at clozapine treatment initiation.

**Results:**

Age at clozapine initiation (Exp(B)=1.098; p<0.001), family history of diabetes (Exp(B)=2.299; p=0.049) and birth weight^2^ (Exp(B)=0.999; p=0.013) were significant predictors of glucose dysregulation onset, while gender was not (Exp(B)=0.1.350; p=0.517). Among individuals with 10 years of follow-up, 80% of those with both abnormal birth weight and a family history of diabetes developed diabetes compared with 56% with only abnormal birth weight, 40% with only a family history of diabetes and 20% in those with neither.

**Conclusions:**

Since 48% of cases had at least one risk factor and 6% had both risk factors, there is a substantial proportion of patients for whom preventive strategies could be implemented.

**Significance of this study**

**What is already known about this subject?**

- Diabetes is over represented in patients with schizophrenia.

**What are the new findings?**

- The new finding is that both abnormal birth weight and first-degree family history of diabetes are independent predictors of diabetes onset in clozapine-treated population.
- Having the two risk factors is strongly associated with diabetes development onset after 10 years of treatment.
- This is very relevant as it could accelerate the implementation of preventive pharmacological strategies.
- Evaluation of these easily accessible risk factors should be embedded in routine clinical practice.

**How might these results change the focus of research or clinical practice?**

- Early pharmacological interventions might be available for a subgroup of patients.

## Introduction

The prevalence of type 2 diabetes mellitus (T2DM) in people with schizophrenia is twice that in the general population^1^ and is a major contributor to the reduced lifespan in this group.^2^ There are no risk factors for glucose dysregulation specific to this population, limiting our ability to identify high-risk individuals and to use preventive strategies. This population might also be exposed to medications with high potential to induce T2DM but to which there is no alternative. Clozapine is the only licensed medication for treatment-resistant schizophrenia, a lifelong medication,^3^ making the need for risk predictors for developing T2DM even more pressing in this group.

Most of the known risk factors for diabetes in people with schizophrenia on antipsychotic medication are the same as those in the general population, such as age,^4^ weight gain^5 6^ and a family history of diabetes.^7^ While specific genetic risk variants for T2DM have been suggested,^8^ such data are not yet available in routine clinical care. Antipsychotic use confers an additional risk that is mediated through an effect on body weight and a direct effect on glucose regulation.^9^ The association between antipsychotic use and weight gain is robust, with up to 75% of patients gaining weight on many of these drugs,^10^ thus limiting the usefulness of body mass index (BMI) and weight gain for risk stratification.

Two clinical factors have been implicated in diabetes risk in both the general population and in patients with schizophrenia, although research in the latter group is more limited. The first of these is a parental history of diabetes. The association between schizophrenia and familial diabetes was first described by Henry Maudsley^11 12^ (*‘* *diabetes is a disease which often shows itself in families in which insanity prevails’*) and has been replicated since.^13 14^ While parental diabetes increases the risk of diabetes in individuals with schizophrenia, this has not been specifically examined in the treatment-resistant group, who are at higher risk due to their long-term use of glucoregulation-altering agents such as clozapine.^7^

The second factor is abnormal birth weight. A recent meta-analysis with over four million individuals showed a 50% increase in T2DM onset in those with abnormal birth weight,^15^ defined as either too low (<3000 g) or too high (>4500 g). The U-shaped risk curve adds evidence to the hypothesis of the fetal origin of the metabolic syndrome.^16^ This hypothesis postulates that intrauterine adversity triggers various adaptations in anticipation of similar conditions in postnatal life,^17^ sparing vital organs such as the brain at the expense of other organs such as the pancreas. This might be protective for immediate postnatal survival and remains so if the environment is similar,^18^ but when the postnatal environment does not match the predicted one these adaptations increase the risk of development of obesity, diabetes and hypertension. Adverse scenarios for these adaptations include the widespread availability of calorie-rich food, or perhaps antipsychotic-induced increased appetite and energy intake.^19^ We have previously reported an inverse correlation between birth weight and increased adiposity in clozapine-treated patients,^20^ and more recently that birth weight modulates weight gain in drug-naïve patients with first-episode psychosis and after clozapine initiation.^21^ To date, there are no published studies on whether abnormal birth weight modulates diabetes onset in patients with schizophrenia.

We hypothesized that in schizophrenia, abnormal birth weight and parental diabetes are independent risk factors for diabetes onset. To test this hypothesis, we examined clozapine-treated patients, as this population shows the highest incidence of diabetes,^22^ using survival analyses accounting for the age of initiation of clozapine and the duration of clozapine treatment.^23^

## Methods

### Design

This was a cross-sectional, single-center study of a clozapine-treated cohort at Cambridgeshire and Peterborough NHS Foundation Trust in Cambridge, UK. Data were collected from anonymized electronic clinical records for the period 2013–2018.

### Sample

In 2013–2018 there were 224 patients with schizophrenia who were on treatment with clozapine. Of these, 27 were excluded due to missing key data for metabolic assessments, date of diagnosis of glucose dysregulation, or date of last primary care blood measurements. A further seven cases were excluded as their diagnosis of T2DM predated clozapine treatment. Of the remaining 190 individuals, data on family history of T2DM were available for 183, birthweight data were available for 112, and data on both these variables were available for 109 individuals. The data are available to other researchers on request.

### Database validity and assessments

The clozapine research database collects and stores clinical information that is routinely sent in clinical letters to the patient and to their general practitioner (GP). Clinical information is collected from both patients and their families. As all patients are on long-term follow-up, the iteration of communications between the psychiatrist, the GP and the patient ensures updating of information and minimizes the risk of errors being perpetuated in the database. The database contains more than 100 clinical variables per assessment and was accessed in May 2018 for the data included in this study.

### Study variables

For the survival analysis, data were censored at the point of diagnosis of glucose dysregulation (either glucose intolerance or T2DM) in the primary care record. The following variables were extracted or derived from the database: (1) paternal and maternal history of diabetes; (2) birth weight (in grams); (3) gender; (4) age; (5) parental age at the time of the patient’s birth; (6) duration of clozapine treatment at the point of censoring; (7) presence or absence of glucose dysregulation as per primary care records; (8) date of diagnosis of glucose dysregulation in primary care; and (9) date of the last recorded blood monitoring in primary care.

Glucose dysregulation (which included glucose intolerance) was defined as the main outcome variable, rather than a more selective outcome of T2DM. This was driven by the clinical relevance of both conditions, given the likely progression from glucose intolerance to diabetes.^24^

Birth weight was routinely collected as part of clinical practice. Birth weight was only included if verified by a parent or if the patient reported the same birth weight at a minimum of two separate consultations, which has been reported as a reliable strategy for collecting this information.^25^ Gestational age at the time of delivery was not routinely collected and was therefore not available for this study. Birth weight was used as a continuous variable and as a categorical variable. For the descriptive statistics and figures, the following categories were used^15^: ≤2500 g (extremely low), 2501–3000 g, 3001–3500 g, 3501–4000 g, 4001–4500 g and >4500 g (high). For birthweight analyses, there were two categories: a high-risk category that comprised the ≤3000 g and >4500 g groups (abnormal birth weight), as both have been linked to increased risk for diabetes onset in the general population, and a low-risk category (3001–4500 g).^15^

### Statistical analyses

For the primary survival analysis, the dependent variable was the duration from (1) initiation of clozapine to either (2a) development of glucose dysregulation or (2b) the last recorded metabolic measures in the primary care record. To specifically examine the role of paternal and maternal history of T2DM, separate binary predictor variables were used for maternal and paternal diabetes.

Kaplan-Meier survival analyses with log-rank tests were used for categorical analyses, comparing the presence or absence of family history of diabetes or abnormal birth weight.

For the analysis of abnormal birth weight as a risk factor and for interactions of birth weight and family history, Cox proportional hazards regression was employed. Age at clozapine initiation, length of clozapine treatment, and gender were used as predictors. Given the known U-shaped relationship between birth weight, we included birth weight as a quadratic variable. The exponentiated model coefficient (e^B^) is presented as HR and its 95% CI. Birth weight and its quadratic value were used in 100 g units.

Descriptive statistics and subgroup differences were examined using χ^2^ tests, *t* tests, and analysis of variance as appropriate. All analyses were conducted using IBM SPSS V.25.0 for Mac, with the significance level set at alpha=0.05.

## Results

The final sample included 190 clozapine-treated patients with schizophrenia. Of these, data on family history of T2DM were available for 183, birthweight data for 112 and data on both variables for 109. The characteristics of the sample are summarized in table 1.

**Table 1:** Sociodemographic and clinical variables for the total study sample and the subgroup with available birthweight information

	Total	Birthweight subgroup
n	190	112
Age in years	48.4 (10.4)	47.1 (9.8)
Gender male	151 (79.8%)	87 (77.7%)
Age at clozapine initiation (in years)	31.7 (8.95)	30.7 (8.24)
Years on clozapine	16.5 (7.0)	16.4 (6.95)
On clozapine for >10 years	144 (75.8%)	83 (74.1%)
On clozapine for >20 years	67 (37%)	40 (39%)
Presence of any glucose dysregulation	47 (24.7%)	33 (29.5%)
Presence of type 2 diabetes mellitus	32 (16.8%)	23 (20.5%)
Family history of diabetes	35 (19%)	23 (21.1%)
Paternal diabetes	18 (9.8%)	12 (11%)
Maternal diabetes	19 (10.4%)	13 (12%)
Paternal age	30.8 (7.5)	30.5 (7.5)
Maternal age	27.2 (5.8)	26.9 (5.4)
Birth weight (g)		3431 (722)
Abnormal birth weight (yes/no)		37 (33%)

Variables are expressed as mean and SD, or number and percentage in brackets.

To test for potential biases, we compared those without birthweight data (n=78) with those with abnormal birth weight (n=75) and normal birth weight (n=37) and found no differences in terms of age at the time of the study (p=0.108), age at clozapine initiation (p=0.051), length of clozapine treatment (p=0.34), family history of diabetes (p=0.68) or gender (p=0.76). Details of these comparisons can be found in the online supplementary material.

10.1136/bmjdrc-2019-001036.supp1 Supplementary data

### Prevalence of glucose dysregulation in direct group comparisons

The prevalence of any glucose dysregulation in the entire sample (n=183) was 24.7% (16.8% for T2DM). The prevalence increased with the duration of clozapine treatment, from 5% in those with less than 5 years on clozapine, to 31% after 10–20 years and 28% after more than 20 years (*F*_1,187_=5.097, df=2, p=0.007; linear-term *F*_1,2_=7.240, df=1, p=0.008).

In the group with family history data (n=183), the prevalence of glucose dysregulation was numerically greater in those with family history of diabetes compared with those without (28.3% vs 16.1%), but this difference was not statistically significant (χ^2^=3.315; df=1; p=0.057).

**Figure 1:**
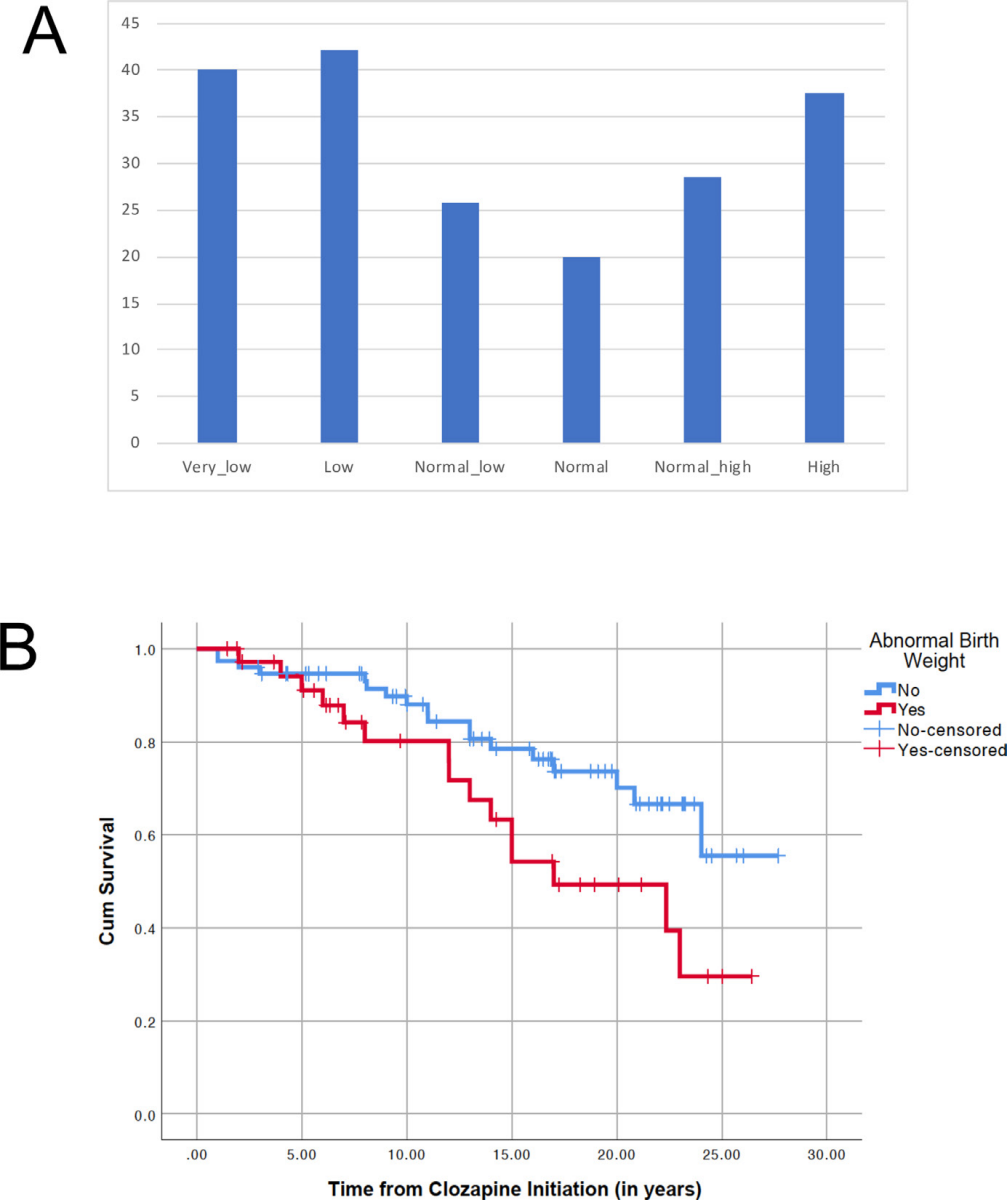
Effect of birth weight on glucose dysregulation onset in clozapine-treated patients. (A) Percentage of any glucose dysregulation by birthweight category in the 112 clozapine-treated cases. (B) Kaplan-Meier survival plot for glucose dysregulation onset using 112 cases, of which 37 had abnormal birth weight (in red; 15 events, 22 censored) and 75 had normal birth weight (in blue; 18 events, 57 censored).

The prevalence of glucose dysregulation varied across the birthweight categories, as shown in figure 1A. Of the 112 individuals, 37 were in the high-risk birthweight categories: <2501 g (n=10), 2501–3000 g (n=19), and >4500 g (n=8). The prevalence of glucose dysregulation was 45.5% in this high-risk subgroup, compared with an overall prevalence of 27.8% in the other three non-high-risk categories, but this difference did not reach statistical significance (χ^2^=3.262; df=1; p=0.058).

**Figure 2:**
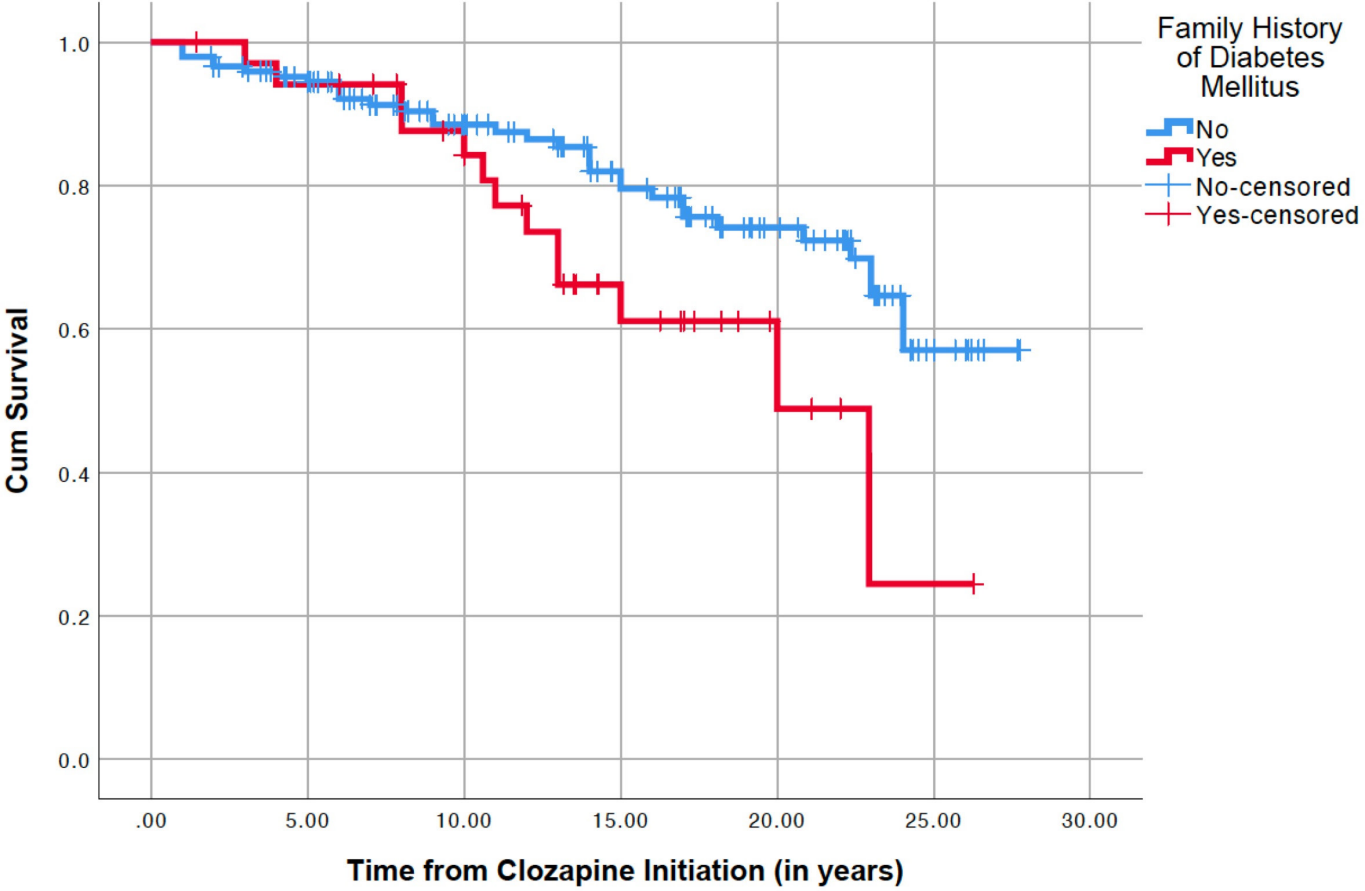
Kaplan-Meier survival plot of glucose dysregulation onset, using 183 cases, of which 148 had no family history of diabetes (in blue; 33 events, 115 censored) and 35 had family history of diabetes (in red; 13 events, 22 censored).

### Role of parental diabetes, controlled by confounders

In the Kaplan-Meier survival analysis (n=183) accounting for length of treatment with clozapine, a greater incidence of glucose dysregulation was seen among those with a parental history of diabetes (Mantel-Cox log-rank χ^2^=4.331; p=0.037; figure 2).

The Cox regression analysis (n=183) showed that parental diabetes (HR=2.145; CI 1.116 to 4.121; p=0.022) and age at clozapine initiation (HR=1.05; CI 1.015 to 1.087; p=0.005) were significant predictors, while gender was not (HR=0.658; CI 0.328 to 1.319; p=0.238). When we examined the role of paternal and maternal diabetes separately, only paternal diabetes (HR=2.751; CI 1.246 to 6.071; p=0.012) and age of clozapine initiation (HR=1.048; CI 1.013 to 1.085; p=0.007) were significant predictors, and not maternal diabetes (HR=1.358; CI 0.571 to 3.229; p=0.489) or gender (HR=0.629; CI 0.313 to 1.266; p=0.194).

### Role of birth weight, controlled by confounders

Using the Kaplan-Meier survival analysis (n=112), a greater incidence of glucose dysregulation was seen in the high-risk birthweight group (Mantel-Cox log-rank χ^2^=4.565; p=0.033; figure 1B).

For the Cox proportional hazards regression survival analysis (n=112), the quadratic element of birth weight was used to account for the U-shaped risk. Birth weight^2^ (HR=0.999; CI 0.998 to 1.000; p=0.026) and age of clozapine initiation (HR=1.092; CI 1.045 to 1.142; p<0.001) were significant predictors, while gender was not (HR=1.167; CI 0.507 to 2.686; p=0.716).

### Exploring the independence of birth weight and family history and alternative models

A Cox proportional hazards regression survival model (n=109) showed that age at clozapine initiation (HR=1.098; CI 1.051 to 1.149; p<0.001), parental history of diabetes (HR=2.299; CI 1.002 to 5.272; p=0.049) and birth weight^2^ (HR=0.999; CI 0.998 to 1.000; p=0.013) were all significant, while gender was not (HR=0.517; CI 0.545 to 3.346; p=0.517).

Birth weight did not differ between individuals with and without a family history of diabetes (mean 3478 g, SD 829.1 vs mean 3429 g, SD 704 respectively; t=−0.288; df=107; p=0.77). Birth weight, or its quadratic value (birth weight^2^), did not correlate with age at clozapine initiation (n=112; r=0.073, p=0.44 and r=0.097, p=0.31, respectively).

To examine if these predictors interacted in determining the risk of glucose dysregulation, we created the following interaction terms: (1) parental diabetes:birth weight^2^; (2) parental diabetes:age of clozapine initiation, and (3) birth weight^2^:age of clozapine initiation. We ran separate models including each of the interaction terms and compared them with the basic model above. None of these models provided better model fits than the basic model (omnibus tests of model coefficients: p=0.908, p=0.548 and p=0.728, respectively), that is, no interaction was found between variables.

### Effect of extreme birth weight and family history on diabetes onset

**Figure 3:**
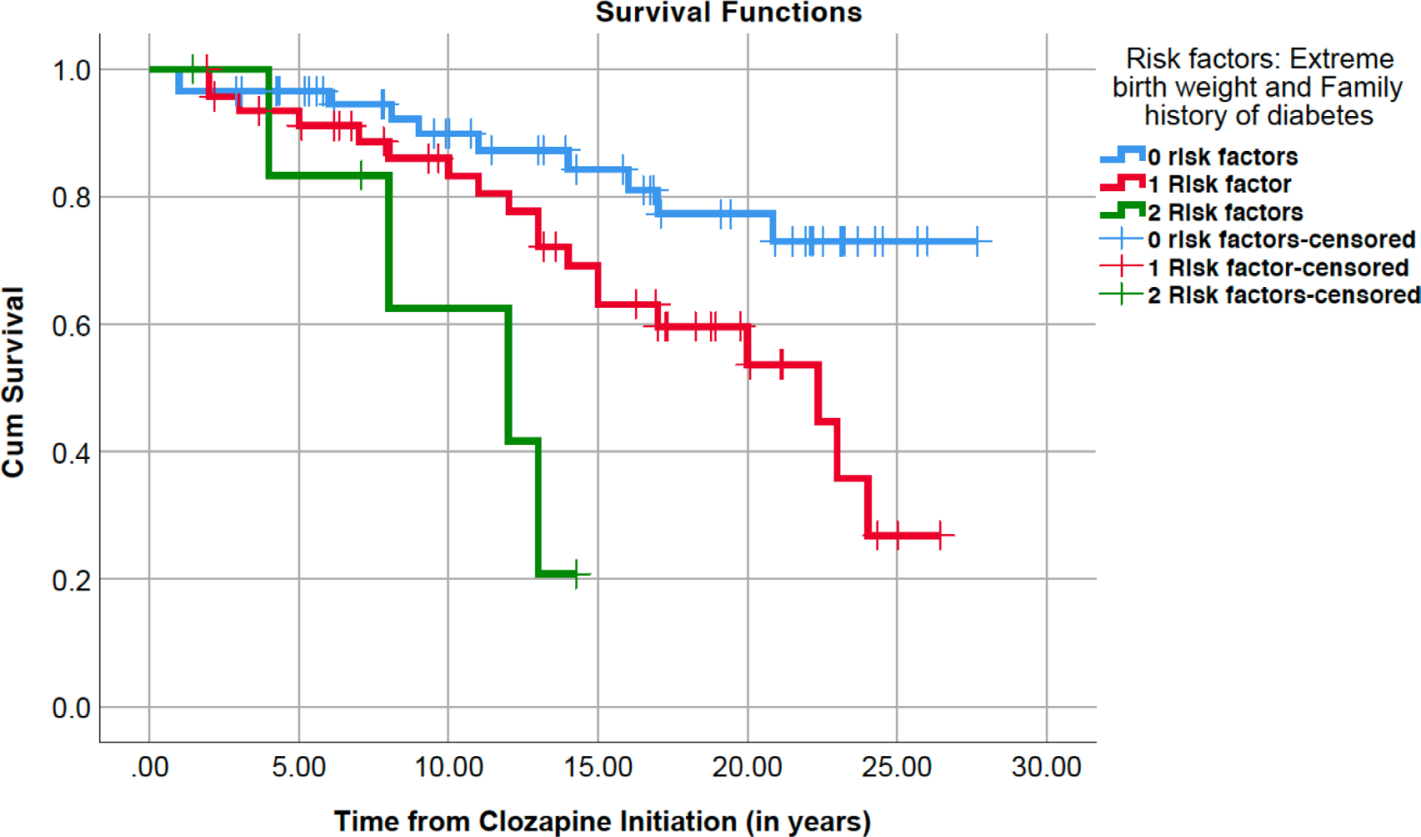
Role of abnormal birth weight and/or family history of diabetes in glucose dysregulation onset using Kaplan-Meier survival plot of 112 cases, of which 58 had no risk factors (in blue; 10 events, 48 censored), 47 had one risk factor (in red; 19 events, 28 censored) and 7 had both risk factors (in green; four events, 3 censored).

In the sample, 58 subjects (51.8%) had none of these two risk factors, 47 (42%) had one and 7 (6.3%) had both risk factors. Glucose dysregulation was seen in 17.2% of those without risk factors, compared with 40.4% of those with one risk factor and 57.1% of those with two risk factors (χ^2^=9.467, p=0.009; linear-by-linear association=9.301, p=0.002). Using the Kaplan-Meier survival analysis, a greater and earlier incidence of glucose dysregulation was seen among those with two risk factors compared with one risk factor or none (Mantel-Cox log-rank χ^2^=14.118; p=0.001; figure 3).

Finally, we explored a clinical question of the risk of developing diabetes in patients with zero, one, or two risk factors depending on age at clozapine initiation (see table 2). For this analysis, we limited the data to those who had been on clozapine for at least 10 years, to exclude individuals who might have not yet developed diabetes during the relatively short follow-up in this sample.

**Table 2:** Risk of developing diabetes by number of risk factors and age at clozapine initiation (less than 25 years old, between 25 and 35, and above 35 years old)

	All ages (%)	<25 (%)	25–35 (%)	>35 (%)
No risk factors (n)	20	12	27	18
Family history of diabetes (n)	40	23	33	75
Extreme birth weight (n)	56	30	43	90
Family history and abnormal birth weight (n)	80	50	100	100

Data only include cases with more than 10 years of clozapine treatment.

## Discussion

In this study, we found that both abnormal birth weight and parental history of diabetes were associated with diabetes onset in clozapine-treated patients with schizophrenia. Both were independent risk factors, which did not interact but had an additive effect. Among those patients treated with clozapine for a decade, up to 80% of cases with both factors and half of those with one factor developed glucose dysregulation. The results cannot be attributed to differences in length of clozapine treatment, gender, or age of clozapine initiation, as these factors were controlled using the Cox regression survival analysis. Considering that 48% of all cases had at least one risk factor and 6% had both risk factors, and should our results be replicated, there might be a substantial proportion of patients for whom strategies to reduce the risk of developing diabetes could be implemented.

Our sample is comparable with other studies in schizophrenia, in terms of its male predominance (~80%), with the prevalence of diabetes (16.8%) and glucose dysregulation (24.7%) being twice that in the general population.^9^ The prevalence found is in line with other studies in clozapine-treated patients.^23^ We excluded individuals with a prior diagnosis of diabetes and those for whom the date of diagnosis was not available; including the latter would have raised the prevalence to 21%. Our sample included individuals who had a very long exposure to clozapine (37% had been on clozapine for over 20 years; table 1). We combined longitudinal assessment in the clinic with patients’ primary care records to increase the completeness and reliability of the information and reduce the false negative rate.

The results replicate and extend others’ work. Family history of diabetes is a risk factor for diabetes onset in the general population^26^ and in people with schizophrenia.^7 14^ A recent meta-analysis has calculated the risk to be fourfold in those with a family history of diabetes, similar to what is found in the general population.^27^ Interestingly, we found that paternal diabetes conferred a greater risk for developing glucose dysregulation. This is in line with findings in first-episode psychosis by our group in Spain^14^ and in schizophrenia conducted in the USA^7^ and the Netherlands,^28^ which suggest a specific role of paternal diabetes in this patient group that has not been described in the general population.^29^ One potential explanation would be that fathers tend to be older than mothers, and therefore more likely to have developed diabetes, but in our sample the paternal and maternal age differed by just 3 years, which makes this explanation unlikely. The consistency of the finding regarding gender-specific family risk along different studies and phases of illness deserves further study. We also replicated the linear association of length of antipsychotic treatment and age at diabetes onset found in schizophrenia.^23^

A more novel part of our study is the role of abnormal birth weight. Compared with previous studies^20^ that have examined the impact of low birth weight exclusively, we considered the impact of both extremes of birth weight and we also used birth weight as a continuous variable. Recent meta-analytic evidence^15^ using more than four million general population cases shows that both low (<3000 g) and very high (>4500 g) birth weight increase the risk of diabetes onset. Our results mimic the same U-shaped curve (figure 1A), with increased risk for extremely low, low, and extremely high birth weights. While there are no previous studies on the effect of birth weight on diabetes onset in people with schizophrenia, the results align with previous work on birthweight modulation of weight gain in schizophrenia,^20 30^ a metabolic complication commonly associated with diabetes. Importantly, the present study adds clinical significance, as here we used well-established markers of glucose dysregulation (diabetes or pre-diabetic state).

The excess of diabetes seen in patients with schizophrenia predates the antipsychotic era,^31^ although the prevalence has increased since the use of second-generation antipsychotics.^5^ Glucose intolerance has also been described in drug-naïve first-episode psychosis,^32 33^ suggesting that some patients might have an innate predisposition, although there are no studies associating these earlier abnormalities with the development of overt clinical conditions such as diabetes. Epidemiological evidence from the famine studies in the Netherlands and China has linked diabetes and schizophrenia onset with low birth weight,^34 35^ reflecting a common developmental origin and risk factor. We think that our study adds evidence to this hypothesis.

The mechanism by which extreme birth weight increases the risk of diabetes onset is still unclear,^16^ and our work here was not aimed to address this. The leading hypothesis in metabolic science, first developed by Hales and Barker,^17^ posits that in-utero adversity leads to epigenetic changes that allow the fetus to cope with a predicted adverse environment by prioritizing energy storage. In a postnatal environment with abundance of resources, these adaptations are disadvantageous and lead to metabolic complications. Schizophrenia is, at least in part, a neurodevelopmental condition^36^ which might predispose to metabolic complications,^37^ and this predisposition is perhaps unmasked by the effects of antipsychotic medication in increasing appetite,^5^ changes in diet^19^ and decrease in resting metabolic rate.^38^ Nonetheless, it might also be interesting to see whether or not the risk factors found here could be replicated in other neurodevelopmental conditions with increased risk of developing metabolic complications, such as attention-deficit/hyperactivity disorder,^39^ or in individuals with schizophrenia taking other antipsychotic medication.

Most importantly, our results provide support for the use of these simple clinical variables to identify cases that will benefit from early preventive metabolic interventions. In this sense, the additive effect of risk factors might have some implications. Having abnormal birth weight and a positive family history (seen in 6% of cases) was almost certainly associated with glucose dysregulation (table 2). A practical consequence would be considering whether hypoglycemic pharmacological treatment in this group is advisable. Indeed, there is a case for adding metformin to the treatment of people on clozapine, both to help reduce weight gain^40^ and because of the high risk of developing diabetes,^41^ after or in conjunction with behavioral changes. Considering that trials for behavioral changes fail to show long-term efficacy,^42^ a pragmatic approach would be to suggest adding metformin to all high-risk cases (ie, those with abnormal birth weight and family history). For cases with one risk factor, lowering the threshold of metformin initiation to those with pre-diabetes might also be advisable. Nevertheless, further studies to replicate our findings should be conducted before making firm recommendations.

Our work has some strengths but also limitations. We used survival analysis, controlling for key factors such as length of clozapine treatment or age at the time of first clozapine prescription, and we included subjects with very long exposure to clozapine. However, the study used birth weight and family history information collected from patients and relatives, which might lack accuracy. We put in place conditions for the validity of the information and we and others have used a similar approach before,^20^ but studies using recorded rather than recalled data might be needed. The group with birthweight data had exactly the same amount of time on clozapine as those without birthweight data (~16 years on average), although they had started 3 years younger. We also defined glucose dysregulation (which included glucose intolerance) as the main outcome, rather than a more selective outcome of T2DM. This was in part driven by the clinical relevance of both conditions, given the likely progression from glucose intolerance to diabetes,^24^ and the sample size of our study. Studies using larger data sets will be needed to replicate our results in those with T2DM only. Sample size in general was limited, with just 112 cases in which birth weight was available, and thus large samples should be used to replicate our findings. Prospective studies and different statistical methods could also be designed to explore causality rather than the association as we found here. Nevertheless, we would like to highlight that schizophrenia is relatively uncommon compared with T2DM (0.7% vs 15% of the general population) and no more than 15% of patients with schizophrenia are treated with clozapine, which inevitably makes studies of clozapine-treated patients smaller compared with studies of T2DM in the general population. We did not include information regarding BMI, as data on BMI at baseline or at the time of diabetes diagnosis were not available, or the rate of BMI increase. However, BMI only marginally increases the risk of diabetes in clozapine patients,^4^ and more importantly we have previously described how BMI is modulated by birth weight in clozapine-treated patients^20^ and in first-episode psychosis. Therefore, the common risk factor (abnormal birth weight) would impact both BMI and glucose dysregulation. Finally, we did not address the issue of psychiatric comedication in this group, which might have had an impact on the development of glucose dysregulation. The sample size and complex medication regimens during the long follow-up (40% more than 20 years) made this infeasible in our study and perhaps could be addressed with larger samples.

In summary, we conclude that abnormal birth weight and family history of diabetes are independent risk factors for glucose dysregulation onset in clozapine-treated patients. Should these results be replicated, it might help clinicians to identify high-risk subjects easily and implement preventive interventions, including the use of pharmacological agents along with changes in lifestyle.
