# Supplementary material for: Birth weight, family history of diabetes and diabetes onset in schizophrenia
Source: BMJ Open Diabetes Res Care. 2020 Jan 28;8(1):e001036. doi: 10.1136/bmjdrc-2019-001036 (PMC7039608; doi:10.1136/bmjdrc-2019-001036)
Supplement: Supplementary data [file bmjdrc-2019-001036supp001.pdf]

Supplementary material. Comparison of the groups to assess for potential bias in the missing birth data group.

	**Subjects without birth weight data**	**Subjects with abnormal birth weight**	**Subjects without abnormal birth weight**	**Statistic and P significance**
N	78	37	75	
Age in years	50.3 (11.1)	46.9 (10.4)	47.1 (9.5)	F(2,187)= 2.255; p=.108
Gender male (n, %)	64 (82.1%)	29 (78.4%)	58 (77.3%)	X^2=.556; p=.757
Age at clozapine initiation (in years)	33.6 (9.7)	31.9 (8.8)	30.0 (7.9)	F(2,187)=3.017; p=.051
Years on clozapine	16.7 (7.1)	15.1 (7.2)	17.1 (6.7)	F(2,187)=1.071; p=.345
Presence of any glucose dysregulation*	14 (17.9%)	15 (40.5%)	18 (31.9%)	**X^2=.6.916; p=.0.031**
Family history of diabetes	12 (16.2%)	7 (19.4%)	16 (21.9%)	X^2=.775; p=.679

*this comparison was expectedly different between groups, as includes a risk factor (abnormal birth weight) and its outcome (glucose dysregulation). When comparing those without birth weight data and those without abnormal birth weight, no differences were found (X^2=.846; p=.358).
